# Very high carriage of gametocytes in asymptomatic low-density *Plasmodium falciparum* and *P. vivax* infections in western Thailand

**DOI:** 10.1186/s13071-017-2407-y

**Published:** 2017-10-24

**Authors:** Wang Nguitragool, Ivo Mueller, Chalermpon Kumpitak, Teerawat Saeseu, Sirasate Bantuchai, Ritthideach Yorsaeng, Surapon Yimsamran, Wanchai Maneeboonyang, Patiwat Sa-angchai, Wutthichai Chaimungkun, Prasert Rukmanee, Supalarp Puangsa-art, Nipon Thanyavanich, Cristian Koepfli, Ingrid Felger, Jetsumon Sattabongkot, Pratap Singhasivanon

**Affiliations:** 1 Department of Molecular Tropical Medicine & Genetics, Faculty of Tropical Medicine, Mahidol University, Bangkok, Thailand; 2 Barcelona Centre for International Health Research, Barcelona, Spain; 3 Population Health & Immunity Division, Walter & Eliza Hall Institute, Parkville, VIC Australia; 4 Department of Medical Biology, University of Melbourne, Parkville, VIC Australia; 5 Mahidol Vivax Research Unit, Faculty of Tropical Medicine, Mahidol University, Bangkok, Thailand; 6 Department of Tropical Hygiene, Faculty of Tropical Medicine, Mahidol University, Bangkok, Thailand; 7 Department of Medical Parasitology and Infection Biology, Swiss Tropical & Public Health Institute, Basel, Switzerland

**Keywords:** Malaria, Asymptomatic infection, Gametocytes, Transmission, Thailand

## Abstract

**Background:**

Low-density asymptomatic infections of *Plasmodium* spp. are common in low endemicity areas worldwide, but outside Africa, their contribution to malaria transmission is poorly understood. Community-based studies with highly sensitive molecular diagnostics are needed to quantify the asymptomatic reservoir of *Plasmodium falciparum* and *P. vivax* infections in Thai communities.

**Methods:**

A cross-sectional survey of 4309 participants was conducted in three endemic areas in Kanchanaburi and Ratchaburi provinces of Thailand in 2012. The presence of *P. falciparum* and *P. vivax* parasites was determined using 18S rRNA qPCR. Gametocytes were also detected by *pfs25 / pvs25* qRT-PCRs.

**Results:**

A total of 133 individuals were found infected with *P. vivax* (3.09%), 37 with *P. falciparum* (0.86%), and 11 with mixed *P. vivax/ P. falciparum* (0.26%). The clear majority of both *P. vivax* (91.7%) and *P. falciparum* (89.8%) infections were not accompanied by any febrile symptoms. Infections with either species were most common in adolescent and adult males. Recent travel to Myanmar was highly associated with *P. falciparum* (OR = 9.0, *P* = 0.001) but not *P. vivax* infections (*P* = 0.13). A large number of *P. vivax* (71.5%) and *P. falciparum* (72.0%) infections were gametocyte positive by *pvs25/pfs25* qRT-PCR. Detection of gametocyte-specific *pvs25* and *pfs25* transcripts was strongly dependent on parasite density. *pvs25* transcript numbers, a measure of gametocyte density, were also highly correlated with parasite density (*r* ^2^ = 0.82, *P* < 0.001).

**Conclusions:**

Asymptomatic infections with *Plasmodium* spp. were common in western Thai communities in 2012. The high prevalence of gametocytes indicates that these infections may contribute substantially to the maintenance of local malaria transmission.

**Electronic supplementary material:**

The online version of this article (10.1186/s13071-017-2407-y) contains supplementary material, which is available to authorized users.

## Background

The last 30 years have seen a great reduction in the burden of malaria in South East Asia in general, and particularly in Thailand [1]. As a consequence of sustained control and economic development in many rural areas, the incidence of malaria has dropped from a peak of 10.7 cases/1000 in early 1981 to currently less than 1/1000. Malaria has been eliminated from most areas of Thailand except the border regions with Myanmar in the west and Cambodia in the east [2, 3]. The reductions have been more pronounced for *P. falciparum* than for *P. vivax*, which now accounts for most local cases in Thailand [1]. The advent of artemisinin resistance on the Thai-Cambodian border [4] and more recently in western Thailand [5, 6], has emphasized the need to move from control to elimination of malaria in these areas [7, 8]. Despite this renewed focus, there is still a substantial lack of understanding on how transmission in the remaining endemic areas is sustained. While Thailand has an excellent system that detects, treats and monitors clinical malaria cases even in relatively remote rural areas [9], there is no routine monitoring of the infectious reservoir.

Low density, asymptomatic infections with *Plasmodium* spp. are common in low endemicity areas worldwide [10–14]. Studies in migrant populations in Thailand have also detected high rates of asymptomatic infections [15, 16]. However, community-based studies are scarce [17–19] and, except for one recent study [20], were small and assayed relatively small volumes of blood from dried filter paper blood spots. More studies with higher detection sensitivity are thus urgently needed to quantify the asymptomatic reservoir of *P. falciparum* and *P. vivax* infections in Thai communities.

Little is known about the role of asymptomatic infections in sustaining local malaria transmission in Thailand. In a single study conducted in Mae Hong Son, it was found that blood from 15.4% and 60% of study participants with afebrile *P. falciparum* and *P. vivax* infections could infect mosquitos [21]. In this study 46% of all microscopy-positive individuals were afebrile, and the number of submicroscopic infections is unknown. Given the prevalence of such afebrile infections and the low incidence rate of clinical malaria, it was concluded that asymptomatic infection constituted the main reservoir of transmission in this population [21].

Light microscopy (LM) is relatively insensitive for detecting gametocytes (and thus potentially infectious parasite carriers), particularly in asymptomatic individuals with low-density infections [22]. In Thailand, gametocytes were more commonly observed in *P. vivax* compared to *P. falciparum* patients [23]. In membrane feeding studies using south-east Asian mosquito *Anopheles dirus*, *P. falciparum* gametocyte observation by LM was strongly linked to *Anopheles dirus* infectivity; only a few patient blood samples without LM-detectable gametocytaemia were found to infect mosquitoes [24, 25]. In contrast, *P. vivax* appears to be more infectious, and a gametocyte density under LM limit of detection can readily lead to mosquito infection [26].

Molecular techniques are much more sensitive at detecting gametocytes than LM [22]. In a study from Thailand that compared LM with molecular methods to detect gametocytes in patients from Tak Province, the proportion of gametocyte positive infections increased from 8.9 to 89.5% for *P. falciparum* and from 31.1 to 91.1% for *P. vivax* [27]. To date, there are no data on the prevalence of gametocytes by PCR in community surveys in Thailand.

We, therefore, conducted a cross-sectional survey of 4309 people living in three malaria endemic communities in Kanchanaburi and Ratchaburi provinces. We combined full population sampling with the sensitive molecular diagnosis of both asexual blood-stage parasites and gametocytes.

## Methods

### Description of study sites

Three sites on a 250 km central span of the Thai-Burmese border were selected for our cross-sectional survey: (i) Suan Phueng district of Ratchaburi Province; (ii) Ban Kong Mong Tha, Sangkhla Buri district of Kanchanaburi Province; and (iii) Ban Bong Ti, Sai Yok district of Kanchanaburi Province. All areas had stable populations (main ethnic groups Thai and Karen) with less than 2% reporting to have travelled in the previous month. Agriculture, farming and forest foraging were main occupations. The annual peak malaria season of these areas was April-July [28].

The southernmost study site, Suan Phueng district, is located 163 km west of Bangkok and encompasses the mountainous regions of the Tanaosri Mountain range. This study site was composed of four connected small villages: Wangko, Huai Krawan, Pong Hang and Huai Phak. The combined population size was 2188. Most houses were clustered along a stream. *Anopheles minimus* and *Anopheles maculatus*, exophagic and nighttime feeders, were the primary malaria vectors in this area. A large-scale cross-sectional survey conducted in 2003–2004 reported monthly prevalence rates of 0.1–1.5% for *P. falciparum* and 0.2–0.6% for *P. vivax* by microscopy [28].

The central site, Bong Ti, was composed of three villages: Ban Bong Ti Bon, Ban Bong Ti Lang and Ban Thai Muang. The combined population size was 3176. The site was situated 65 km west of Kanchanaburi in hilly terrain. To date, no detailed malaria epidemiological and entomological study has been conducted in this area. Most houses were accessible by paved roads. The northernmost site Kong Mong Tha was a single village located in a remote valley surrounded by mountains. The population size was 959. Once accessible only by boats or foot for 10 months of the year, the village had become accessible by trucks throughout the year at the time of this study. Previous studies done in 2000 estimated malaria prevalence to be 5.2% for *P. falciparum* and 5.9% for *P. vivax* by LM and identified the predominant anophelines as *A. minimus*, *A. sawadwongporni*, *A. maculatus*, *A. campestris* and *A. barbirostris* [29].

### Survey methods

Participation in the study was household based. Convenience sampling of households was used, and all household members were invited to participate. The expected sample sizes, i.e. the total residents in the participating households, were 1858 (Suan Phueng), 3034 (Bong Ti), and 678 (Kong Mong Tha). Informed consent was obtained from each participant and, when needed, a legal guardian. Demographic characteristics and behavioural risk data were obtained by trained interviewers using a structured questionnaire. Before blood collection, body temperature was measured with an infrared thermometer. Participants who had body temperature > 37.5 °C received a rapid diagnosis test (SD BIOLINE Malaria Ag P.f/Pan, Standard Diagnostics, Gyeonggi-do, Republic of Korea) and, if positive, were referred for treatment at the nearest malaria clinic. In this study, “fever” was defined as body temperature > 37.5 °C, and “unwell” as any feeling of illness reported by the participants including but not limited to chills, nausea, headache, muscle pain and abdominal pain.

### Sample collection, DNA and RNA extraction

Capillary blood (250 μl) was collected by finger prick from each participant into an EDTA-containing microtainer. A 50 μl portion was immediately preserved for RNA extraction onsite by mixing with 250 μl RNAprotect (Qiagen, Hilden, Germany) and transferred to -20 °C for long-term storage within 12 h. The remaining blood was separated into pellet and plasma on the same day in a temporary field laboratory and stored at -20 °C. If the finger prick blood was < 250 μl, the volume of the draw was recorded.

After sample shipment on dry ice to Faculty of Tropical Medicine, Mahidol University, Bangkok, DNA was purified from each blood pellet (≤ 100 μl) with FavorPrep 96-Well Genomic DNA Extraction Kit (Favorgen, Ping-Tung, Taiwan) at following manufacturer’s protocol. Purified DNA was eluted in 100 μl Elution Buffer and stored at -20 °C. Two samples of *P. falciparum* NF54 strain (100 μl packed cells with 0.01% ring stage parasitemia) were used as DNA extraction controls for every 81 survey samples. RNA was extracted with RNeasy Plus Mini kit (Qiagen). In addition to the proprietary gDNA elimination step of the kit, on-column treatment with RNase-Free DNase set (Qiagen) was used on all samples to further remove trace genomic DNA contaminant.

### Detection of parasite DNA and gametocyte-specific transcripts by qPCR

All qPCR and qRT-PCR measurements were performed on a CFX96 Realtime PCR Detection System and analysed with CFX Manager Software Version 3.0 (Bio-Rad Laboratories, California, USA). All qPCRs used i*Taq* Universal Probes Supermix (Bio-Rad Laboratories), and all qRT-PCRs used Superscript III One-Step Quantitative RT-PCR System (Invitrogen, Massachusetts, USA). Copy number was estimated using in-plate standards generated from a serial dilution of a plasmid containing an appropriate DNA fragment. The performance of each assay can be found in Additional file 1: Table S1. Detection limits were assessed by 10-fold dilution series performed in 5 replicates. The limit of detection was defined as the lowest plasmid copy number at which detection was successful in > 50% of the replicates.

Malaria parasite infection was first identified by the QMAL assay, a genus-specific probe-based qPCR targeting a conserved region in 18S rRNA genes of plasmodia [30], using 4 μl of purified DNA which were equivalent to 8 μl of whole blood. If positive by QMAL, 4 μl DNA was subjected to species-specific qPCR assays to identify and quantify *P. falciparum* and *P. vivax* as previously described [31] except for a new forward primer (5′-TTG TTA CTT TGA GTA AAA TTA AGT GTT CAT AAC-3′) for *P. falciparum*.

Gametocyte carriage was inferred from the presence of *pfs25* or *pvs25* transcripts. Four microliters of purified RNA were used in a single-step qRT-PCR for *pfs25* or *pvs25* [30]. Two quality assurance assays were applied to all RNA samples. First, QMAL was run to ensure that there was no genomic DNA contamination. Secondly, a single-step qRT-PCR using QMAL primers and probe was applied to all RNA samples; only samples positive for 18S rRNA transcripts were analysed for gametocytes.

### Statistical analyses

Standard methods for the analyses of epidemiological data were used. A chi-square test was used to determine the statistical significance of the difference between proportions. Multivariate analysis of factors associated with parasite carriage was conducted using logistic regression. Association between gametocyte and parasite densities was investigated by linear regression. All analyses were performed using Stata 13 (Stata Crop. College Station, TX) or R version 2.14.0 (https://CRAN.R-project.org).

## Results

### Demographic characteristics

In September and October 2012, a total of 4309 individuals were surveyed: 2359 in Bong Ti, 415 in Kong Mong Tha and 1535 in Suan Phueng. These represented 74.3, 70.2 and 43.3% of the population in each site respectively; 51.6% of participants were female, and all but 6 (0.14%) had resided in the village for longer than 2 months. Children aged 1–6 and 7–12 years accounted for 17.6 and 19.7% of participants, respectively. 9.5% were adolescents (13–17 years), and 39.4% and 13.8% were adults aged 19–49 and 50+, respectively. Only 78 participants (1.8%) reported travel outside the village in the previous month, with 27 of these having visited Myanmar. 90.5% of participants reported using a bednet last night with most (80.5%) owning a net for more than 2 years.

### Prevalence of *P. falciparum* and *P. vivax* infections

A total of 180 participants were found to be infected with either *P. vivax* or *P. falciparum* (overall prevalence 4.18%): 133 *P. vivax* (*Pv*: 3.09%), 37 *P. falciparum* (*Pf*: 0.86%) and 11 mixed *P. vivax/ P. falciparum* (0.26%) infections.

**Fig. 1:**
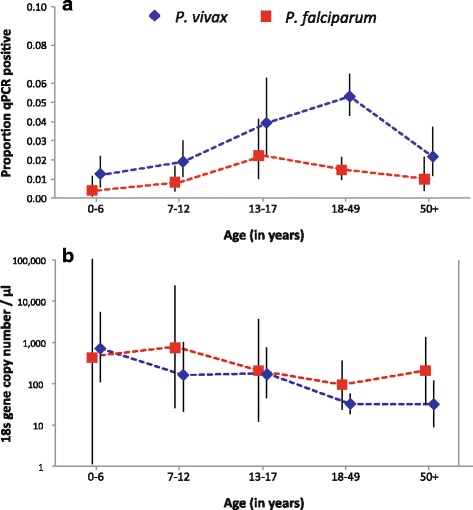
Parasite prevalence rates (**a**) and geometric mean parasite densities (**b**) as a function of age. Whiskers denote 95% confidence intervals

Similar levels of infections were found in Bong Ti (*Pv*: 3.82%; *Pf*: 1.36%) and Suan Phueng (*Pv*: 3.12%; *Pf*: 1.17%; Chi-square test, *df* = 1; *Pv*: *χ* ^2^ = 1.29, *P* = 0.26; *Pf*: *χ* ^2^ = 0.33, *P* = 0.57 and Fisher’s exact test, *P* > 0.26). However, in Kong Mong Tha only 6 *P. vivax* (1.45%, Chi-square test: *df* = 1, *Pv*: *χ* ^2^ = 5.11, *P* = 0.024) and no *P. falciparum* infections (Fisher’s exact test: *P* = 0.012) were detected. Infections with either *P. vivax* or *P. falciparum* were significantly more common in male participants (Chi-square test: *df* = 1; *Pv*: *χ* ^2^ = 24.6, *P* ≤ 0.001; *Pf*: *χ* ^2^ = 9.75, *P* = 0.002) and varied among age groups (Table 1; *Pv*: Chi-square test: *χ* ^2^ = 34.1, *df* = 4, *P* ≤ 0.001; *Pf*: Fisher’s exact test: *P* = 0.017) peaking in adolescents for *P. falciparum* (2.20%) and adults for *P. vivax* (5.30%, Fig. 1a).

**Table 1:** Prevalence rates (PR) and multivariate predictors of infection with *P. vivax* and *P. falciparum* by qPCR. Adjusted odds ratios (AOR), 95% confidence intervals (95% CI) and *P*-values are given only for statistically significant factors

		*P. vivax*				*P. falciparum*			
	*n*	PR	AOR	95% CI	*P*-value	PR	AOR	95% CI	*P*-value
Region									
Bong Ti	2359	3.82		1.85–3.80	0.016	1.36			
Kong Mong Tha	415	1.45	0.36	0.16–0.83		0			
Suan Phueng	1535	3.12	0.69	0.47–1.01		1.17			
Gender									
Female	2222	2.03			< 0.001	0.63			0.003
Male	2087	4.74	2.65	1.85–3.80		1.72	2.63	1.40–4.94	
Age group (years)									
< 7	758	1.19			< 0.001	0.40			0.088
7–12	849	1.88	1.87	0.82–4.29		0.82	2.51	0.67–9.88	
13–17	409	3.91	3.93	1.71–9.04		2.20	5.91	1.56–22.4	
18–49	1699	5.30	5.70	2.83–11.5		1.47	3.78	1.12–12.8	
≥ 50	594	2.19	2.09	0.88–4.97		1.01	2.74	0.67–11.2	
Recent travel									
No	4231	3.29				1.09			
Yes	78	6.41				5.13			
To Myanmar	27	7.41				11.11	9.04	2.53–32.2	0.001
Feeling unwell									
No	4103	3.12			0.001	1.12			
Yes	206	7.77	3.56	2.03–6.58		1.94			
Malaria last 2 weeks									
No	4289	3.36				1.05			< 0.001
Yes	19	0				26.32	30.1	9.87–92.0

Participants that reported recent travel were more likely to be infected but this only reached statistical significance for *P. falciparum* (3.85 *vs* 1.06%, Chi-square test: *χ* ^2^ = 5.38, *df* = 1, *P* = 0.020) and not *P. vivax* (Chi-square test: *χ* ^2^ = 2.32, *df* = 1, *P* = 0.13). Remarkably, 4 of 27 people (14.8%) that travelled to Myanmar the previous month had *Plasmodium spp*. infections (2 *Pf*, 1 *Pv*, 1 *Pf* + *Pv*). The use of a bednet was, however, not associated with a reduction in risk of infection for either *P. vivax* (prevalence in users: 3.37% *vs* non-users: 3.18%; Chi-square test: *χ* ^2^ = 0.04, *df* = 1, *P* = 0.83) or *P. falciparum* (1.18 *vs* 0.73%, Fisher’s exact test: *P* = 0.62). Five of the 19 participants (26.3%, Fisher’s exact test: *P* < 0.001) that reported having had a malaria episode in the last 2 weeks were infected with *P. falciparum*, none with *P. vivax*.

The majority of *P. vivax* (91.7%) and *P. falciparum* (89.8%) infections were not accompanied by any febrile symptoms. However, participants with either *P. vivax* and *P. falciparum* infections were nevertheless significantly more likely to report having febrile symptoms in the previous 2 days than uninfected individuals (*Pv*: 8.3 *vs* 4.0%, Chi-square test: *χ* ^2^ = 6.75, *df* = 1, *P* = 0.009; *Pf*: 10.0 *vs* 4.0%, Fisher’s exact test, *P* = 0.035). Participants with *P. vivax* more commonly reported feeling ‘unwell’ at the time of the surveys (11.1 *vs* 4.6%, Chi-square test: *χ* ^2^ = 13.1, *df* = 1, *P* < 0.001).

In multivariate analyses, male gender, age, feeling unwell at the time of the survey and area of residence remained independently associated with the risk of *P. vivax* infection (Table 1). Male gender, age, recent travel to Myanmar and malaria episode in last 2 weeks were independent predictors of *P. falciparum* infections.

### *Plasmodium* spp. densities

Most infections were of very low densities with estimated geometric mean 18S rDNA copy numbers of 8.5/μl (95% CI: 5.6–12.7) and 22.9/μl (95% CI: 8.9–59.5) for *P. vivax* and *P. falciparum*, respectively.

*Plasmodium vivax* parasite densities decreased significantly with age (Fig. 1b, ANOVA: *F*_(4,135)_ = 4.56, *P* = 0.002) and were higher in people reporting feeling “unwell”: 37.3 (95% CI: 14.3–97.3) *vs* 7.0 (95% CI: 4.6–10.9) 18S copies/μl; ANOVA: *F*_(1,142)_ = 6.73, *P* = 0.011). The low number of *P. falciparum* infections precluded an analysis of factors associated with *P. falciparum* parasite densities.

### Gametocyte prevalence

**Fig. 2:**
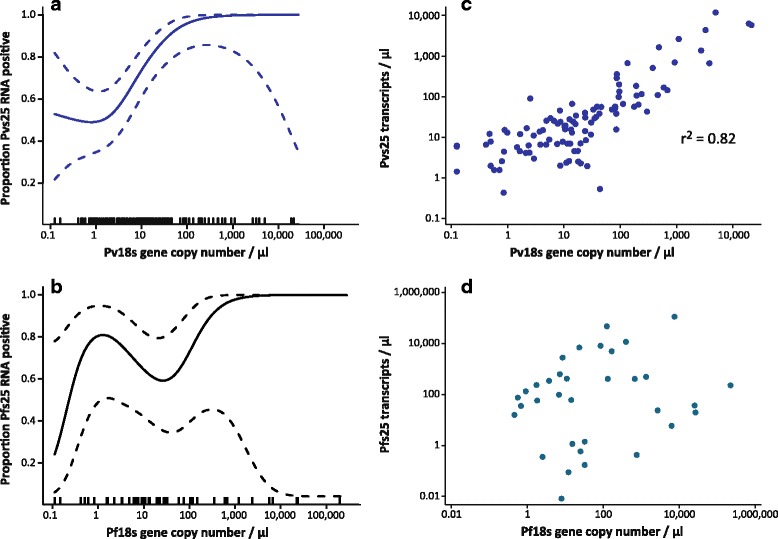
Relationships between parasite density and presence of gametocytes. **a**, **b** Relationships between parasite density (*pv18S* or *pf18S* gene copy number/μl) and gametocyte positivity. **c**, **d** Relationship between parasite density and gametocyte density (*pvs25* or *pfs25* transcripts/μl)

Overall, 103 of 144 *P. vivax* (71.5%) and 36 of 50 *P. falciparum* (72%, *P* = 0.94) infections were positive respectively for *pvs25* and *pfs25* by qRT-PCR, resulting in a population prevalence of 2.40% *P. vivax* and 0.84% *P. falciparum* gametocytemic individuals. The presence of *pvs25* and *pfs25* transcripts in a sample was dependent on parasite density (Fig. 2a, b): with each 10-fold increase in parasitaemia the odds of detecting gametocytes increased 3.14-fold (95% CI: 1.85–5.33; Likelihood-ratio (LR) test: *χ* ^2^ = 24.1, *df* = 1, *P* < 0.001) for *P. vivax* and 1.83-fold (95% CI: 1.01–3.32; Likelihood-ratio (LR) test: *χ* ^2^ = 5.2, *df* = 1, *P* = 0.045) for *P. falciparum*, respectively. No other variables were significantly associated with the detection of *P. vivax* or *P. falciparum* gametocytes.

On average 26.3 (geometric mean, 95% CI: 17.6–39.2) *pvs25* and 57.8 (95% CI: 16.0–208.3) *pfs25* transcripts/μl were detected in *P. vivax* and *P. falciparum* gametocyte positive samples, respectively. *pvs25* transcript numbers were highly correlated with parasite density as gauged by the 18S rDNA copy numbers (Fig. 2c, *r* ^2^ = 0.82, *P* < 0.001). No significant correlation was found between *pfs25* transcript- and 18S rDNA copy numbers (Fig. 2d, *r* ^2^ = 0.18, *P* = 0.32).

## Discussion

As a result of the sustained Thai malaria control programs that is based on clinical case management and vector controls over the last decades, the burden of malaria in Thailand has progressively been reduced, restricting transmission mostly to the national borders with Myanmar and Cambodia [1]. The Thai malaria surveillance system, however, is entirely based on detection of clinical cases and does not detect asymptomatic *Plasmodium* spp*.* infections. Our study filled this gap in knowledge. Using sensitive molecular techniques, we measured the prevalence of *P. falciparum* and *P. vivax* together with their gametocyte prevalence in three populations on the western border of the country.

We found that *Plasmodium* infections in Bong Ti and Suan Phueng had an overall prevalence rate of 4.2%. These two sites accounted for 90% of the participants in this study. Because both sites were part of larger communities and were located on the hilly terrain occupied primarily by agricultural Thai and Karen residents, they should be representative of many areas along Thailand western border. On the other hand, Kong Mong Tha was a small village geographically isolated from the nearest populous areas. It thus represented a unique setting where *P. falciparum* appeared to have been eliminated.

Because a portion of very low-density infections inevitably has escaped detection, the prevalence likely was underestimated. Recently, Imwong and coworkers used an “high-volume” 18S rRNA qPCR to determine the distribution of parasite density in endemic populations of northwestern Thailand and western Cambodia [32]. The distribution was found to be unimodal and Gaussian after log-transformation. Assuming the same parasite density distribution and the detection limit of ~ two parasites/μl for our protocol [30, 33], we would have underestimated the true prevalence of *Plasmodium* by approximately two-fold.

We observed a decline in *P. vivax* parasitemia as age increased (Fig. 1b). This trend extended into adulthood and was much more gradual than that previously found in Papua New Guinea (PNG), in which parasitemia peaked in the youngest stratum (0–3 years) and rapidly declined in the older age groups [34]. This difference may reflect the lower endemicity of the current study site (3 *vs* 13% in PNG) where acquired immunity against the parasite likely took longer to develop.

A previous study conducted in Suan Phueng estimated the prevalence of *Plasmodium* spp. infections by light microscopy to be 0.1–2.1% in 2004 and showed a higher prevalence of *P. falciparum* than *P. vivax* [28]. In the present survey, 3.9% of participants were PCR-positive, predominantly with *P. vivax*. It is difficult to directly compare the prevalence in both surveys because different diagnoses were used. Interestingly, we found that Hamlet 2, a village in Suan Phueng which previously had distinctly high prevalence, still had the highest prevalence in 2012, its value exceeding twice the average of the other villages (data not shown). This village, unlike others, also saw no decrease in parasite prevalence during the 1994–2004 control program. Thus, it appears that this village was a local transmission hotspot that needed special attention by the malaria control program.

Comparing our current results to those from 2004 in Suan Phueng, the age structure of both *P. vivax* and *P. falciparum* infections appears to have shifted towards adults, suggesting reduced risks of malaria transmission within the household and a relative increase in occupation-related outdoor inoculations. This may reflect the effectiveness of the local indoor residual spraying and bednet donation programmes. However, the shift may be explained by improved diagnosis, which allowed better detection of low parasitemia infections in adults [34, 35].

The overall prevalence of *Plasmodium* infections of 4.9% in Bong Ti is comparable to that observed (5.7% by PCR) in a small active case detection survey conducted in the same district during the high transmission peak in June 2012 [18].

Interestingly, 14.8% (4/27) of people from Suan Phueng and Bong Ti who reported to have travelled to Myanmar during the previous month had infections. Of these, three had *P. falciparum* or mixed infections. Given with the overall low prevalence of *P. falciparum* infections (0.9%), this suggests that a large portion of the *P. falciparum* infections may be imported by people who travelled across the border.

We only identified six infected individuals in Kong Mong Tha, all of whom had *P. vivax* and none had *P. falciparum*. The overall prevalence by PCR (0.6%) was much lower than the average LM prevalence of 10% in 2000–2001 [29]. Thus, despite the sensitive molecular diagnosis used, fewer infections were detected in the current study. The marked decline in parasite prevalence coincided with improved infrastructure of the village, health management, and accessibility by motor vehicles. As expected from its advantageous biology for transmission [36], *P. vivax* appears to be more resilient than *P. falciparum*.

Most infections were of low density, and only 10% of infections were accompanied by febrile symptoms. This suggests that, despite low transmission levels in recent years, residents still acquire and maintain a substantial level of clinical immunity to *Plasmodium* spp. infections. Because 10% (5/50) of all *P. falciparum* infections were detected within 2 weeks after a previous malaria episode, these infections may not truly represent asymptomatic infections, but rather, symptomatic infections after anti-malarial treatment. Thus, the proportion of non-febrile symptoms at the time of survey may have slightly underestimated the risk of symptomatic infection.

*pvs25* and *pfs25* transcripts were found in a large proportion of asymptomatic infections, indicating a potentially large contribution to the transmission reservoir. We found that both the probability of detecting *pvs25* transcripts and density were strongly correlated with the 18S rDNA copy number, a proxy for circulating parasite density. While such a correlation had been reported previously for clinical cases [37, 38], our study demonstrated that this relationship also holds true for low-level asymptomatic infections. We also found a similar relationship in a recent cross-sectional survey in Papua New Guinea [34]. The tight association (*r* ^2^ = 0.82) between *pvs25* transcript and 18S rDNA copy numbers will permit future estimation of the gametocyte reservoir in the population from blood DNA without costly qRT-PCR.

In contrast, we found no correlation between *pfs25* transcript abundance and *P. falciparum* parasitemia. This may be a result of the slow development of *P. falciparum* gametocytes, which takes approximately 2 weeks, and their longevity [22]. In other words, *P. falciparum* gametocyte density may be correlated with parasitemia during the preceding weeks but not at the time of blood sampling. In addition, because of cytoadherence and parasite sequestration, the total *P. falciparum* load may not be faithfully reflected in the peripheral blood sampled. The faster development of the *P. vivax* gametocyte, its shorter life-span, and the presence of all blood stages in the circulation may together permit the detection of a positive relationship between the copy numbers of *pvs25* transcripts and 18S rDNA.

Two studies recently reported the relationship between gametocytemia and infection of *An. dir*us, a major local vector of southeast Asia. These studies demonstrated that robust mosquito infection by *P. vivax* requires only a few gametocytes/μl blood [26] while infection by *P. falciparum* requires ~100 gametocytes/μl [25], suggesting that low-density asymptomatic infections may contribute more to *P. vivax* than to *P. falciparum* transmission in the population. Using the parasite density distribution in Thailand and Cambodia [32], we previously argued that asymptomatic *P. vivax* carriers contribute significantly to transmission [26]. Because asymptomatic infection allows the parasite to be transmitted without the antimalarial drug pressure from treatment, this may be one factor that underlies the slow emergence of drug resistance in *P. vivax* compared to *P. falciparum* in the area.

The high proportion of asymptomatic, gametocyte positive infection presents a significant challenge to the malaria control programme that is based exclusively on case management and vector control. Our data thus suggest that all infected individuals, particularly those with *P. vivax*, should be considered transmission-competent. The lack of an association of bednet use with risk of either *P. vivax* or *P. falciparum* infection indicates that current vector control methods may have reached the limit of effectiveness. A novel approach to target the large reservoir of asymptomatic parasites may thus be required if Thailand’s goal of malaria elimination in 2025 is to be achieved.

## Conclusion

Asymptomatic infections were common in endemic areas of western Thailand in 2012. Most infections were associated with gametocytemia and thus may contribute to onward transmission. Targeting these silent transmission reservoirs will be an important task for Thailand’s malaria elimination program.

## Additional file

Additional file 1: Table S1. Performance of qPCR and qRT-PCR. The threshold cycles (C_T_) are shown for detection of plasmid standards at different copy numbers per reaction. The means and the standard errors of the mean (SEM) are shown for C_T_ values used to determine the amplification efficiency (E) and *r* ^2^, with values in parenthesis excluded. *Neg* indicates no amplification. The limit of detection (red) is defined as the lowest copy number with > 50% success rate. (DOCX 24 kb)

## Abbreviations

LM: Light microscopy
qPCR: Quantitative real-time polymerase chain reaction
qRT-PCR: Quantitative reverse transcription polymerase chain reaction
rDNA: Ribosomal DNA
rRNA: Ribosomal RNA
