# Supplementary material for: Very high carriage of gametocytes in asymptomatic low-density Plasmodium falciparum and P. vivax infections in western Thailand
Source: Parasit Vectors. 2017 Oct 24;10:512. doi: 10.1186/s13071-017-2407-y (PMC5655986; doi:10.1186/s13071-017-2407-y)
Supplement: Additional file 1: Table S1. — Performance of qPCR and qRT-PCR. The threshold cycles (CT) are shown for detection of plasmid standards at different copy numbers per reaction. The means and the standard errors of the mean (SEM) are shown for CT values used to determine the amplification efficiency (E) and r 2, with values in parenthesis excluded. Neg indicates no amplification. The limit of detection (red) is defined as the lowest copy number with > 50% success rate. (DOCX 24 kb) [file 13071_2017_2407_MOESM1_ESM.docx]

**Additional file 1: Table S1**. Performance of qPCR and qRT-PCR. The threshold cycle (C_T_) are shown for detection of plasmid standards at different copy numbers per reaction. The means and the standard errors of the mean (SEM) are shown for C_T_ values used to determine the amplification efficiency (E) and r^2^, with values in parenthesis excluded. *Neg* indicates no amplification. The limit of detection (red) is defined as the lowest copy number with > 50% success rate.

**A: QMAL** (E = 104.5%, r^2^ = 0.989)

| **plasmid copies** | **1. C_T_** | **2. C_T_** | **3. C_T_** | **4. C_T_** | **4. C_T_** | **5. C_T_** | **Mean C_T_** | **SEM** |
| --- | --- | --- | --- | --- | --- | --- | --- | --- |
| **4,000,000** | 19.68 | 19.91 | 20.26 | - | - | - | 19.95 | 0.17 |
| **400,000** | 22.70 | 22.74 | 23.03 | - | - | - | 22.82 | 0.10 |
| **40,000** | 26.02 | 26.04 | 26.23 | - | - | - | 26.10 | 0.07 |
| **4,000** | 29.22 | 29.26 | 29.29 | 29.41 | 29.41 | 29.42 | 29.32 | 0.04 |
| **400** | 32.03 | 32.26 | 32.51 | 32.60 | 32.60 | 33.00 | 32.48 | 0.16 |
| **40** | 34.78 | 34.90 | 35.98 | 36.48 | 36.48 | 37.82 | 35.99 | 0.56 |
| **20** | 35.05 | 35.46 | *Neg* | *Neg* | *Neg* | *Neg* | - | - |
| **16** | 35.03 | 36.67 | 36.98 | 38.52 | 38.52 | *Neg* | - | - |
| **12** | 36.14 | 36.59 | 36.75 | 41.41 | 41.41 | *Neg* | - | - |
| **4** | **36.59** | **37.28** | **38.89** | ***Neg*** | ***Neg*** | ***Neg*** |  |  |
| **0** | *Neg* | *Neg* | *Neg* |  | *Neg* | *-* | - | - |

**B: *Pf18S*** (E = 96.2%, r^2^ = 0.990)

| **plasmid copies** | **1. C_T_** | **2. C_T_** | **3. C_T_** | **4. C_T_** | **5. C_T_** | **Mean C_T_** | **SEM** |
| --- | --- | --- | --- | --- | --- | --- | --- |
| **40,000** | 24.53 | 24.58 | 24.73 | - | - | 24.61 | 0.06 |
| **4,000** | 27.72 | 28.05 | 28.35 | - | - | 28.04 | 0.18 |
| **400** | 31.32 | 31.71 | 31.74 | - | - | 31.59 | 0.14 |
| **40** | 34.07 | 34.43 | 34.58 | 35.51 | 35.65 | 34.85 | 0.31 |
| **20** | 35.10 | 36.15 | 36.24 | 38.05 | *Neg* | - | - |
| **16** | 35.33 | 36.29 | 37.23 | 38.53 | *Neg* | - | - |
| **12** | **36.29** | **37.57** | **44.97** | **46.85** | ***Neg*** | - | - |
| **8** | 35.46 | 36.36 | *Neg* | *Neg* | *Neg* | - | - |
| **4** | 35.52 | 43.43 | *Neg* | *Neg* | *Neg* | - | - |
| **0** | *Neg* | *Neg* | *Neg* | - | - | - | - |

**C. *Pv18S*** (E = 91.4%, r^2^ = 0.986)

| **plasmid copies** | **1. C_T_** | **2. C_T_** | **3. C_T_** | **4. C_T_** | **5. C_T_** | **Mean C_T_** | **SEM** |
| --- | --- | --- | --- | --- | --- | --- | --- |
| **400,000** | 22.29 | 22.61 | - | - | - | 22.45 | 0.16 |
| **40,000** | 25.71 | 25.77 | 25.79 | - | - | 25.76 | 0.02 |
| **4,000** | 29.24 | 29.20 | 29.11 | 29.16 | 29.08 | 29.16 | 0.03 |
| **400** | 32.49 | 33.07 | 33.25 | 32.79 | 32.46 | 32.81 | 0.16 |
| **40** | 36.53 | 38.19 | (44.97) | 36.2 | 35.25 | 36.54 | 0.61 |
| **20** | 37.02 | 35.92 | 35.06 | 37.18 | *Neg* | - | - |
| **16** | 45.22 | 36.96 | 37.09 | *Neg* | *Neg* | - | - |
| **12** | 37.22 | 38.02 | 37.00 | *Neg* | *Neg* | - | - |
| **8** | **38.11** | **37.11** | **36.75** | ***Neg*** | ***Neg*** | - | - |
| **4** | 39.21 | *Neg* | *Neg* | *Neg* | *Neg* | - | - |
| **0** | *Neg* | *Neg* | - | - | *-* | - | - |

**D. *Pfs25*** (E = 90.1%, r^2^ = 0.986)

| **plasmid copies** | **1. C_T_** | **2. C_T_** | **3. C_T_** | **4. C_T_** | **5. C_T_** | **Mean C_T_** | **SEM** |
| --- | --- | --- | --- | --- | --- | --- | --- |
| **40,000** | 24.68 | 24.83 | 25.08 | - | - | 24.86 | 0.12 |
| **4,000** | 28.01 | 28.21 | 28.03 | - | - | 28.08 | 0.06 |
| **400** | 32.14 | 32.01 | (30.33) | - | - | 32.08 | 0.07 |
| **40** | 35.52 | 34.28 | 36.66 | 35.26 | 35.75 | 35.49 | 0.38 |
| **20** | 36.71 | 36.53 | 33.19 | 37.04 | *Neg* | - | - |
| **16** | 40.72 | 35.91 | *Neg* | *Neg* | *Neg* | - | - |
| **12** | **36.57** | **37.74** | **44.84** | **33.22** | ***Neg*** | - | - |
| **8** | 35.81 | *Neg* | *Neg* | *Neg* | *Neg* | - | - |
| **4** | 43.43 | 34.22 | *Neg* | *Neg* | *Neg* | - | - |
| **0** | *Neg* | *Neg* | - | - | - | - | - |

**E*. Pvs25*** (E = 109.7%, r^2^ = 0.983)

| **plasmid copies** | **1. C_T_** | **2. C_T_** | **3. C_T_** | **4. C_T_** | **5. C_T_** | **Mean** | **SEM** |
| --- | --- | --- | --- | --- | --- | --- | --- |
| **40,000** | 24.34 | 24.27 | 24.28 | - | - | 24.30 | 0.02 |
| **4,000** | 27.52 | 27.58 | 27.36 | - | - | 27.49 | 0.07 |
| **400** | 31.04 | 30.85 | 30.26 | - | - | 30.72 | 0.23 |
| **40** | 33.87 | 33.61 | 33.72 | 32.31 | 34.63 | 33.63 | 0.37 |
| **20** | 33.82 | 34.40 | *Neg* | *Neg* | *Neg* | - | - |
| **16** | 33.53 | 40.44 | 36.29 | *Neg* | *Neg* | - | - |
| **12** | **33.60** | **34.76** | **36.23** | ***Neg*** | ***Neg*** | - | - |
| **8** | 35.24 | 35.02 | *Neg* | *Neg* | *Neg* | - | - |
| **4** | 38.98 | 36.50 | *Neg* | *Neg* | *Neg* | - | - |
| **0** | *Neg* | *Neg* | - | - | - | - | - |
